# Self-Reported Discrimination in Health-Care Settings Based on Recognizability as Transgender: A Cross-Sectional Study Among Transgender U.S. Citizens

**DOI:** 10.1007/s10508-017-1028-z

**Published:** 2017-08-07

**Authors:** Amanda Rodriguez, Anette Agardh, Benedict Oppong Asamoah

**Affiliations:** 1 Masters Programme in Public Health, Faculty of Medicine, Lund University, Malmö, Sweden; 2 Social Medicine and Global Health, Department of Clinical Sciences CRC, Lund University, Jan Waldenströms gata 35, House 28, Floor 12, 205 02 Malmö, Sweden

**Keywords:** Transgender, Discrimination, Health care, Sex worker, HIV

## Abstract

Discrimination has long been tied to health inequality. Rejected by families and communities because of their gender identity and gender-role behavior, transgender individuals are often socially marginalized. This study aimed to assess discrimination in health-care settings among persons self-identifying as transgender in the U.S. in relation to their recognizability as transgender, operationalized as how often they experienced that others recognized them as transgender. Data were obtained from the National Transgender Discrimination Survey (*n* = 6106 participants, assigned sex at birth = 3608 males, 2480 females, respectively). Binary logistic regressions were performed to examine associations between transgender recognizability and discrimination in health-care settings. Being recognized as transgender to any extent had a significant effect on perceived discrimination in health care. Always recognized as transgender showed significant associations with discrimination in a health-care setting (OR 1.48) and the following individualized health-care settings: social service settings (rape crisis and domestic violence centers, OR 5.22) and mental health settings (mental health clinic and drug treatment program, OR 1.87). Sex work and other street economy, which are known experiential factors affected by discrimination, were also significantly associated with discrimination in health-care settings. Discrimination in health-care settings is pervasive for transgender who are recognized as transgender. Public health efforts to improve access to equitable health care for transgender individuals may benefit from consideration of demographic, experiential, and medical risk factors to more fully understand the source of the seemingly excess risk of discrimination among persons recognized by others as being transgender.

## Introduction

Discrimination has long been tied to health inequality. The World Health Organization (2001) has linked health disparities to sociostructural inequalities that stem from discrimination in societies. Sexual orientation and gender identity that is other than the heteronormative of most societies have been a target for discrimination (Rivers & D’Augelli, 2001).

It has been argued that the stress of living in a society that devalues one’s identity can lead to adverse health outcomes (Clark, Andersson, Clark, & Williams, 1999). Studies have shown that stress due to discrimination is damaging to mental and physical health (Pascoe & Richman, 2009; Williams & Mohammed, 2009). Transgender individuals are more likely to be victims of hate crimes because of discrimination (Boehmer, Bowen, & Bauer, 2007; National Coalition of Anti-Violence Programs, 2013), and often live with daily threats of violence and face great discrimination in employment, housing, access to education, and health care (Harper & Schneider, 2003). High levels of victimization and discrimination may also lead to internalized transphobia, which can further affect adverse health outcomes.

Transgender is an all-encompassing term used to describe people whose assigned sex at birth differs from their gender identity or expression as well as those who vary from or reject socially/culturally constructed definitions of gender in terms of the female–male dichotomy (Institute of Medicine, 2011). This term covers a wide variety of lifestyles and ideas of self. The transgender community has been an understudied population, and knowledge is lacking as to the extent to which discrimination affects transgender individuals (Institute of Medicine, 2011). The transgender population is extremely small, both internationally and within the U.S., with an estimated 0.3% of U.S. adults identifying as transgender (Gates, 2011). The limited size of the transgender population poses an obstacle for gaining access to survey samples that are large enough for statistical analysis. However, transgender research has become an emerging field, as shown by an increasing number of studies focusing on transgender persons, gender identity, and related issues (for recent reviews, see Dhejne, van Vlerken, Heylens, & Arcelus, 2016; Zucker, Lawrence, & Kreukels, 2016).

Rejected by families and communities because of their gender identity, transgender individuals are often socially marginalized. This may lead to a disproportionate number of homeless and unemployed transgender persons who then enter illegal activities such as sex work or the selling of drugs to gain an income (Galea & Vlahov, 2002; Reisner et al., 2009). A small study by Garofalo, Deleon, Osmer, Doll, and Harper (2006) found that 67% of ethnic minority male-to-female transgender youth had ever been arrested, and 37% had a history of incarceration, i.e., excessively high prevalence for population-level U.S. statistics. Furthermore, in previous studies of transgender, a high prevalence of adverse health outcomes has been found, including HIV infection, substance use, poor mental health, suicidal thoughts and attempts, and exposure to many forms of violence and discrimination (Clements-Nolle, Marx, Guzman, & Katz, 2001; Dhejne et al., 2016; Hotton, Garofalo, Kuhns, & Johnson, 2013; Kenagy, 2005; Nadal, Davidoff, & Fujii-Doe, 2014; Zucker et al., 2016).

Transgender persons often perceive themselves as stigmatized in health-care settings (Hughto, Reisner, & Pachankis, 2015). The factors that might contribute to discrimination in health-care settings are complex and include physicians’ own feelings of uncertainty and lack of cultural competency with regard to transgender persons, perceived discrimination at the systems level despite policy, and care providers’ assumptions that transgender persons may be mentally unstable or have psychiatric issues (Lefkowitz & Mannell, 2017; Snelgrove, Jasudavisius, Rowe, Head, & Bauer, 2012).

No study, to the best of our knowledge, has looked at the extent to which the recognizability of transgender status might affect transgender persons’ perceived discrimination in health-care settings. This research aimed to assess discrimination in health-care settings among those who self-identify as transgender in the U.S. in relation to their recognizability as transgender. It was hypothesized that persons whose transgender status is recognizable to others face greater discrimination when accessing health-care services compared to those whose transgender status is not recognizable. Moreover, in order to examine the possibility that perceived discrimination might vary according to health-care setting due to the type of provider encountered and/or the reason care was sought, a broad range of health-care settings was examined, including social and mental health services. Further, it is increasingly recognized that the various forms of discrimination, such as those due to race/ethnicity, sexual identity, and other personal characteristics, intersect in ways that might reinforce one another. Thus, in addition to transgender recognizability, the study examined selected personal characteristics that might affect perceived discrimination in health-care settings, including assigned sex at birth, ethnicity, involvement in street economy, incarceration, and HIV and disability status. Assigned sex at birth was considered a potential source of discrimination due to gender-related discrimination as well as the greater complexity of sex-reassignment procedures in women. Sexual orientation was also considered as a potential confounder since we also deemed that as a potential source of discrimination. The results of this study may provide a better understanding of the role of transgender recognizability for the delivery of optimal health-care services tailored to the individual’s needs and inform gender-affirming care, cultural competency training efforts, and policies that confront discrimination of transgender individuals.

## Method

### Participants

Data from the National Transgender Discrimination Survey, a large convenience sample of transgender adults, provided the impetus for this study of discrimination in health-care settings faced by transgender persons who are recognizable by others as being transgender. The data originated from a national survey distributed by health and outreach workers organized by the National Gay and Lesbian Task Force and the National Center for Transgender Equality in 2008. The original dataset included 6450 transgender and gender nonconforming study participants who had answered “yes” to the question “Do you consider yourself to be transgender/gender nonconforming in any way?” (A clarification was provided in the questionnaire that “transgender/gender nonconforming” describes people whose gender identity or expression is different, at least part of the time, from the sex assigned to them at birth). The response alternatives were “yes” and “no.” Persons who answered “no” were requested not to continue. If “yes,” participants could further respond to the following questions: “What sex were you assigned at birth on your original birth certificate?” (Response alternatives: “male” or “female”) and “What is your primary gender identity?” (Response alternatives: “male/man,” “female/woman,” “part time as one gender,” “part time as another,” “a gender not listed here, please specify….”). Anyone who reported an assigned sex at birth different from primary gender identity was classified as transgender. Further, those who chose the same gender for both sex at birth and primary gender identity, or who chose the part time or gender not listed here for the latter, were also classified as transgender or gender nonconforming depending on their answer to other questions. Additionally, participants were asked to indicate from among 16 possible sub-categories of gender identity/expression which one(s) they “strongly identified” with. In the full sample, the most frequently endorsed sub-categories were transgender, male-to-female transgender, gender nonconforming, transsexual, and female-to-male transgender. Participants could endorse as many sub-categories as they liked. Transgender individuals from all 50 states, the District of Columbia, Puerto Rico, Guam, and the U.S. Virgin Islands completed online or paper surveys. Surveys were distributed by more than 800 transgender-led or transgender-serving community-based organizations in the U.S. and its territories. The final sample was limited to those who identified themselves as U.S. citizens and for whom information was available on perceived discrimination in health-care settings, i.e., 6106 transgender and gender nonconfirming individuals (assigned sex at birth *n* = 3608 males, *n* = 2480 females).

### Procedure

This study was cross-sectional in design. Survey data were used in order to measure the association between transgender recognizability (predictor variable) and perceived discrimination in health-care settings (outcome variable). The National Transgender Discrimination Survey covered a range of topics evaluating discrimination in all facets of transgender life. In this study, questions that did not address health care were excluded. The final material included data concerning possible confounders that were known to influence transgender discrimination. These were identified from literature reviews and included: assigned sex at birth, ethnicity/race, education, sex work, drug sales, other street economy, having been to jail/prison, gender-related mental health diagnosis, disability, and HIV status. For some questionnaire items, apart from a “yes” or “no” answer, the participant could choose the option “not applicable.” If chosen, that person’s response was not included in the analysis of that item.

### Measures

#### Outcome

Discrimination in a health-care setting was used as the primary outcome variable. This variable was derived from responses to questions regarding experiences of physical abuse, verbal harassment, and/or being denied equal treatment in the following health-care settings: doctor/hospital, emergency room, ambulance/emergency medical technician (EMT), rape crisis center, domestic violence center, mental health clinic, and drug treatment program. The responses were dichotomized as “yes,” indicating experience of one or more forms of discrimination (physical abuse, verbal harassment, and/or denied equal treatment) in at least one of the seven health-care settings mentioned above, and “no,” indicating no experience of discrimination in any of the health-care settings.

The secondary outcome was to investigate discrimination in specific health-care settings. These variables included: discrimination in a social service setting (rape crisis center or domestic violence center, aggregated into one variable) discrimination in a mental health setting (mental health clinic or drug treatment program, aggregated into one variable), and lastly, discrimination in a hospital/doctor, ambulance, emergency room, and emergency medical technician (EMT) setting (aggregated into one variable). These variables were dichotomized as “yes” (experienced discrimination) and “no” (not reported any experience of discrimination) in the specific health-care settings mentioned above.

#### Main Exposure (Determinant)

Being recognized as a transgender/gender nonconforming (GNC) person was the primary exposure. The variable was based on self-report by the participant. This predictor variable was an ordinal categorical variable derived from the response to the statement, “People can tell I am transgender/gender nonconforming even if I don’t tell them.” Response alternatives were: 1 = always, 2 = most of the time, 3 = sometimes, 4 = occasionally, and 5 = never. The reference group was those who reported never being identified as transgender.

#### Covariates

Age was captured by the question that enquired about participant’s current age in years. This was categorized in the analyses as: 18–24 years, 25–34 years, 35–44 years, 45–54 years, and 55+ years.

Assigned sex at birth originated from the question, “What sex were you assigned at birth on your original birth certificate?” The response options were 0 = male and 1 = female. As everyone in the dataset was a transgender person, this variable would show whether assigned sex had an effect.

Ethnicity/race included: White, Black or African American, American Indian or Alaska Native, Hispanic or Latino, Asian or Pacific Islander, Arab or Middle Eastern, and Multiracial or Mixed race. Individual ethnicities/races were coded as follows: 1 = White, 2 = Black/African American, 3 = American Indian or Alaska Native, 4 = Hispanic or Latino, 5 = Asian or Pacific Islander, 6 = Arab or Middle Eastern, and 7 = multiracial or mixed race.

Education was assessed by the question, “Highest education level completed.” This variable was used as a proxy for socioeconomic status (Winkleby, Jatulis, Frank, & Fortmann, 1992). The response options were: 1 = elementary/junior high, to 11 = doctorate degree. The responses were then recoded: 1 = some (elementary/junior high, some high school, high school diploma/GED and <1 year of college), 2 = medium (tech school degree, some college (>1 year), associate degree and bachelor’s degree), and 3 = advanced (master’s degree, professional degree and doctorate degree). Advanced was used as the reference.

Information concerning participation in sex work, drug sales, and other street economy was assessed by the question, “Have you ever worked for pay in the street economy?” The response options were dichotomized as 0 = “no” and 1 = “yes.”

Jail/Prison was assessed by the question, “Have you ever been to jail or prison for any reason?” The response options were “no” and “yes.”

Gender-related mental health diagnosis, i.e., gender identity diagnosis, was assessed by the question, “Have you ever received a gender-related mental health diagnosis?” The response options were “no” and “yes.”

Information concerning disability was assessed by the question, “What is your disability?” The response options were 0 = not selected, or 1 = physical disability, 2 = learning disability, and 3 = mental health disability. The responses to the four options were combined and dichotomized as 0 = “no” and 1 = “yes, disability.”

HIV status was assessed from the question, “What is your HIV status?” The response options were: HIV negative, HIV positive, and Don’t know. HIV negative was the reference.

Sexual orientation was assessed by the question, “What is your sexual orientation?” The response options were: Gay/Lesbian/Same-gender attraction, Bisexual, Queer, Heterosexual, Asexual, and Other, please specify.

### Statistical Analysis

SPSS 22.0 was used for analysis, where statistical significance was determined at the alpha .05 level. Descriptive statistics were used to determine frequencies. Binary logistic regressions were performed to examine associations between transgender recognizability and discrimination in health-care settings. Variables with *p* value >.05 were excluded in the multivariable regression models. Associations were first examined in separate multivariable models where each model was adjusted for a group of confounders with presumed similarity. Demographic variables included age, assigned sex at birth, ethnicity/race, and education. Experiential factors were sex work, drug sales, other street economy, and having ever been to jail/prison. Medical factors included gender-related mental health diagnosis, disability, and HIV status. Models were fit regressing discrimination in health-care settings on recognizability as transgender and controlled for demographics, experiential factors, and medical factors. The final model included all possible confounders.

The original data collection was approved by the Institutional Review Board of Pennsylvania State University.

## Results

A total of 6106 transgender participants were included in the study of which 3608 were assigned “male” at birth accounting for 59.3% of participants. Approximately 49% reported that they were always, most of the time, or sometimes recognized as transgender. The vast majority of participants identified as White (*n* = 4699). Identifying as Black/African American, American Indian/Alaska Native, or Hispanic/Latino was nearly evenly distributed (*n* = 269, *n* = 247, and *n* = 240) and 456 identified as multiracial/mixed race. Approximately 80% had an education higher than a secondary school level. Almost 10% had ever worked in sex work. More than 15% had ever been to jail/prison and 2.4% were HIV positive. In a health-care setting, one-third (33.1%) of transgender individuals had experienced discrimination. Looking further at individual health-care settings, between 4 and 7% had experienced discrimination at a rape crisis center, domestic violence center, and/or a drug treatment program. The settings with the largest amount of discrimination reported were the mental health clinic (14.5%), emergency room (16.8%), and doctor/hospital (28.7%) (Table 1).

**Table 1:** Frequencies of sample characteristics of 6106 transgender persons in the U.S.^a^

Characteristic	Frequency (*n*)	Percent (%)
People can tell I’m trans/GNC		
Always	372	6.1
Most of the time	977	16.1
Sometimes	1639	27.0
Occasionally	1789	29.4
Never	1302	21.4
Sexual orientation		
Gay, lesbian, same-gender attracted	1402	23.4
Bisexual	1420	23.7
Queer, pansexual, non-binary attracted	1419	23.7
Heterosexual	1363	22.7
Asexual	274	4.6
Other	122	2.0
Age		
18–24 years	1026	18.4
25–34 years	1873	33.5
35-–44 years	1009	18.1
45–54 years	943	16.9
55+ years	734	13.1
Assigned sex at birth		
Male	3608	59.3
Female	2480	40.7
Ethnicity/race		
White	4699	77.5
Black/African American	269	4.4
American Indian/Alaska Native	247	4.1
Hispanic/Latino	240	4.0
Asian Pacific/Islander	126	2.1
Arab/Middle Eastern	27	0.4
Multiracial/mixed race	456	7.5
Education		
Low	1198	19.7
Medium	3665	60.2
High	1221	20.1
Sex work (total)	591 (6106)	9.7 (100)
Drug sales (total)	488 (6106)	8.0 (100)
Other street economy (total)	185 (6106)	3.0 (100)
Jail/Prison (total)	947 (6106)	15.6 (100)
Gender-related mental health diagnosis (total)	3093 (6106)	51.0 (100)
Disability (total)	2004 (6106)	32.8 (100)
HIV status		
HIV negative	5415	89.4
HIV positive	146	2.4
I don’t know my status	496	8.2
Experienced discrimination		
In a health-care setting [aggregate^b^] (total)	2022 (6106)	33.1 (100)
Rape crisis center (total^c^)	155 (4588)	5.0 (100)
Doctor/hospital (total^c^)	1658 (5779)	28.7 (100)
Emergency room (total^c^)	773 (4568)	16.8 (100)
Ambulance/Emergency Med. Tech. (total^c^)	246 (3544)	6.9 (100)
Domestic violence center (total^c^)	195 (3131)	6.2 (100)
Mental health clinic (total^c^)	634 (4360)	14.5 (100)
Drug treatment program (total^c^)	135 (3112)	4.3 (100)

^a^The total, *N*, varies from variable to variable because of missing data

^b^Health-care setting is a combination of all the settings: hospital/doctor, emergency room, ambulance/emergency medical technician, rape crisis center, domestic violence shelter, mental health clinic, and drug treatment program

^c^The total excluded those who responded as “not applicable” because they have not tried to access the setting

Table 2 shows the prevalence of experienced discrimination in health-care settings based on transgender recognizability, demographic characteristics, experiential characteristics, and medical characteristics. Prevalence of discrimination was greatest among those reporting recognizability as trans/GNC always and most of the time, i.e., 40.9 and 36.9%, respectively. Among those who had ever worked in sex work, drug sales or other street economy, more than half (57.7, 54.1, and 62.2%) experienced discrimination in a health-care setting. Of the total sample, 3093 (50.7%) had received a gender-related mental health diagnosis, with nearly half (*n* = 1268) of them having experienced discrimination in a health-care setting.

**Table 2:** Prevalence of discrimination in a health-care setting and sample characteristics, including demographics, experiential factors and medical factors, among 6106 U.S. transgender individuals^a^

Characteristic	Experienced discrimination		Chi-square *p* value
	Yes *N* (%)	No *N* (%)	
People can tell I’m trans/GNC			<.001
Always	152 (40.9)	220 (59.1)	
Most of the time	361 (36.9)	616 (63.1)	
Sometimes	486 (29.7)	1153 (70.3)	
Occasionally	592 (33.1)	1197 (66.9)	
Never	419 (32.2)	883 (67.8)	
Sexual orientation			.536
Gay, lesbian, same-gender attracted	460 (32.8)	942 (67.2)	
Bisexual	489 (34.4)	931 (65.6)	
Queer, pansexual, non-binary attracted	457 (32.2)	962 (67.8)	
Heterosexual	443 (32.5)	920 (67.5)	
Asexual	100 (36.5)	174 (63.5)	
Other	45 (36.9)	77 (63.1)	
*Demographics*			
Age			.286
18–24 years	340 (33.1)	686 (66.9)	
25–34 years	606 (32.4)	1267 (67.6)	
35-–44 years	313 (31.0))	696 (69.0)	
45–54 years	325 (34.5)	618 (65.5)	
55+ years	260 (35.4)	474 (64.6)	
Assigned sex at birth			.019
Male	1231 (34.1)	2377 (65.9)	
Female	782 (31.5)	1698 (68.5)	
Ethnicity/race			<.001
White	1458 (31.0)	3241 (69.0)	
Black/African American	90 (33.5)	179 (66.5)	
American Indian/Alaska Native	117 (47.4)	130 (52.6)	
Hispanic/Latino	88 (36.7)	152 (63.3)	
Asian Pacific/Islander	33 (26.2)	93 (73.8)	
Arab/Middle Eastern	11 (40.7)	16 (59.3)	
Multiracial/mixed race	210 (46.1)	246 (53.9)	
Education			.003
Low	367 (30.6)	831 (69.4)	
Medium	1199 (32.7)	2466 (67.3)	
High	451 (36.9)	770 (63.1)	
*Experiential factors*			
Sex work			
Yes	341 (57.7)	250 (42.3)	<.001
No	1681 (30.5)	3834 (69.5)	
Drug sales			
Yes	264 (54.1)	224 (45.9)	<.001
No	1758 (31.3)	3860 (68.7)	
Other street economy			
Yes	115 (62.2)	70 (37.8)	<.001
No	1907 (32.2)	4014 (67.8)	
Jail/Prison			
Yes	415 (43.8)	532 (56.2)	<.001
No	1599 (31.2)	3529 (68.8)	
*Medical factors*			
Gender-related mental health diagnosis			
Yes	1268 (41.0)	1825 (59.0)	<.001
No	750 (25.2)	2227 (74.8)	
Disability			
Yes	701 (35.0)	1303 (65.0)	.030
No	1321 (32.2)	2781 (67.8)	
HIV status			.025
HIV negative	1808 (33.3)	3612 (66.7)	
HIV positive	42 (42.5)	84 (57.5)	
I don’t know my status	151 (30.4)	345 (69.6)	

*p* value = significance level, *p* value <.05 = significant

^a^Health-care setting is a combination of all the settings: hospital/doctor, emergency room, ambulance/emergency medical technician, rape crisis center, domestic violence shelter, mental health clinic and drug treatment program

Table 3 shows the prevalence of discrimination in a health-care setting based on current sexual orientation and being recognized as transgender, stratified by assigned sex at birth. There was a skewed distribution of being recognized as transgender for both birth-assigned males and females but in different directions, with birth-assigned males reporting more frequent transgender recognizability than females. There was a statistically significant relationship between recognizability as trans/GNC and experience of discrimination in a health-care setting only among birth-assigned males. No such relationship was found among birth-assigned females. Also, no statistically significant differences were found in experience of discrimination in a health-care setting based on sexual orientation among both sexes.

**Table 3:** Prevalence of discrimination in a health-care setting, recognizability as transgender and sexual orientation among 6106 U.S. transgender, individuals stratified by assigned sex at birth^a^

Characteristic	Experienced discrimination (birth-assigned male) *N* (%)		Chi-square value, *p* value	Experienced discrimination (birth-assigned female) *N* (%)		Chi-square value, *p* value
	Yes	No		Yes	No	
People can tell I’m trans/GNC			26.04, <.001			3.86, .426
Always	143 (41.6)	201 (58.4)		9 (32.1)	19 (67.9)	
Most of the time	340 (37.2)	574 (62.8)		20 (33.9)	39 (66.1)	
Sometimes	446 (30.0)	1042 (70.0)		36 (24.7)	110 (75.3)	
Occasionally	246 (33.7)	483 (66.3)		342 (32.4)	709 (67.5)	
Never	44 (41.1)	63 (58.9)		375 (31.4)	819 (68.6)	
Sexual orientation			6.12, .294			1.10, .954
Gay, lesbian, same-gender attracted	315 (33.5)	625 (66.5)		144 (31.5)	313 (68.5)	
Bisexual	391 (35.1)	722 (64.9)		96 (31.7)	207 (68.3)	
Queer, pansexual, non-binary attracted	75 (31.0)	167 (69.0)		378 (32.2)	795 (67.8)	
Heterosexual	306 (33.2)	617 (66.8)		136 (31.1)	301 (68.9)	
Asexual	86 (38.7)	136 (61.3)		14 (26.9)	38 (73.1)	
Other	34 (41.5)	48 (58.5)		11 (27.5)	29 (72.5)	

^a^Health-care setting is a combination of all the settings: hospital/doctor, emergency room, ambulance/emergency medical technician, rape crisis center, domestic violence shelter, mental health clinic and drug treatment program

Table 4 shows the results of the bivariate analyses (crude odds ratios) for the relationship between each separate characteristic and discrimination according to health-care setting. Discrimination in a health-care setting was positively associated with the category “always” being recognized as trans/GNC (OR 1.45, 95% CI 1.08, 1.95). Transgender who identified as American Indian/Alaska Native ethnicity/race were about twice as likely (OR 1.82, 95% CI 1.37, 2.40) to experience discrimination in a health-care setting than those who identified as White. Discrimination in a health-care setting was also positively associated with transgender who had received a gender-related mental health diagnosis (OR 2.17, 95% CI 1.89, 2.44). There was a strong association between having experienced discrimination in a health-care setting and having worked in sex work (OR 2.17, 95% CI 2.19, 3.35) and having worked in other street economy (OR 2.56, 95% CI 1.82, 3.58).

**Table 4:** Bivariate analysis of sample characteristics and discrimination in health-care settings among 6106 U.S. transgender individuals, odds ratios (OR), and 95% Confidence Intervals (CI)^a^

	Discrimination in a health-care setting^c^ (*N* = 6106)	Discrimination in rape crisis or domestic violence center (*N* = 3218)	Discrimination in mental health clinic or drug treatment program (*N* = 4404)	Discrimination in hospital/doctor/ambulance or emergency room setting (*N* = 5803)
Characteristic	OR (CI)^b^	OR (CI)^b^	OR (CI)^b^	OR (CI)^b^
People can tell I’m trans/GNC				
Always	1.45* (1.08, 1.95)	4.70* (2.79, 7.92)	1.91* (1.35, 2.70)	1.23 (0.96, 1.57)
Most of the time	1.20 (0.95, 1.52)	2.77* (1.71, 4.48)	1.60* (1.22, 2.10)	1.14 (0.95, 1.36)
Sometimes	0.813 (0.65, 1.00)	2.40* (1.55, 3.71)	1.35* (1.06, 1.73)	0.85 (0.72, 0.10)
Occasionally	0.95 (0.80, 1.13)	1.98* (1.28, 3.07)	1.14 (0.89, 1.45)	0.98 (0.84, 1.15)
Never	1 (ref)	1 (ref)	1 (ref)	1 (ref)
Assigned sex at birth				
Male	1 (ref)	1 (ref)	1 (ref)	1 (ref)
Female	0.89 (0.75, 1.04)	0.60* (0.46, 0.79)	0.76* (0.64, 0.90)	0.94 (0.84, 1.05)
Ethnicity/race				
White	1 (ref)	1 (ref)	1 (ref)	1 (ref)
Black/African American	0.79 (0.57, 1.10)	3.91* (2.65, 5.77)	1.44* (1.01, 2.05)	0.93 (0.70, 1.23)
American Indian/Alaska Native	1.82* (1.37, 2.40)	2.09* (1.22, 3.55)	1.91* (1.35, 2.69)	2.02* (1.55, 2.63)
Hispanic/Latino	1.15 (0.86, 1.55)	2.01* (1.18, 3.42)	1.63* (1.11, 2.38)	1.33* (1.01, 1.76)
Asian Pacific/Islander	0.84 (0.55, 1.28)	0.51 (0.12, 2.09)	1.11 (0.61, 2.01)	0.79 (0.52, 1.21)
Arab/Middle Eastern	1.36 (0.60, 3.09)	5.80* (1.82, 18.44)	2.15 (0.78, 5.96)	1.61 (0.72, 3.59)
Multiracial/mixed race	1.51* (1.22, 1.87)	2.98* (2.02, 4.40)	2.44* (1.87, 3.18)	1.90* (1.56, 2.32)
Education				
Low	0.63* (0.52, 0.76)	1.83* (1.22, 2.73)	1.27 (0.98, 1.65)	0.68* (0.57, 0.81)
Medium	0.76* (0.62, 0.88)	1.28 (0.88, 1.86)	1.20 (0.96, 1.50)	0.80* (0.69, 0.91)
High	1 (ref)	1 (ref)	1 (ref)	1 (ref)
Sex work	2.71* (2.19, 3.35)	7.62* (5.78, 10.04)	3.99* (3.24, 4.92)	2.54* (2.13, 3.02)
Drug sales	1.91* (1.53, 2.38)	4.23* (3.08, 5.82)	3.11* (2.46, 3.94)	2.42* (1.99, 2.93)
Other street economy	2.56* (1.82, 3.58)	5.72* (3.67, 8.93)	3.37* (2.38, 4.78)	3.28* (2.42, 4.44)
Jail/Prison	1.26* (1.07, 1.49)	3.12* (2.38, 4.08)	2.32* (1.92, 2.80)	1.55* (1.34, 1.80)
Gender-related mental health diagnosis	2.17* (1.93, 2.44)	1.53* (1.18, 1.98)	1.73* (1.46, 2.06)	2.04* (1.82, 2.29)
Disability	1.13* (1.00, 1.28)	0.88 (0.67, 1.16)	1.07 (0.90, 1.27)	1.16* (1.03, 1.30)
HIV status				
HIV negative	1 (ref)	1 (ref)	1 (ref)	1 (ref)
HIV positive	0.83 (0.54, 1.26)	3.47* (2.18, 5.52)	2.22* (1.49, 3.29)	1.24 (0.88, 1.76)
I don’t know my status	0.92 (0.74, 1.14)	1.26 (0.80, 2.00)	1.19 (0.89, 1.59)	0.88 (0.71, 1.09)

* Significance at .05 level

^a^The total, *N*, varies from variable to variable because of missing data and also the exclusion of those who responded as “not applicable” because they have not tried to access the setting

^b^OR odds ratio, CI 95% Confidence Interval

^c^Health-care setting is a combination of all the settings: hospital/doctor, emergency room, ambulance/emergency medical technician, rape crisis center, domestic violence shelter, mental health clinic and drug treatment program

Concerning the individualized health-care settings, always being recognized as transgender/GNC had 4.70 (95% CI 2.79, 7.92) times the odds of discrimination in a rape crisis center or domestic violence center and 1.91 (95% CI 1.35, 2.70) times the odds in a mental health clinic or drug treatment program. Ethnicity/race showed positive associations in individualized health-care settings. For example, Arab/Middle Eastern American and Black/African American transgender were over 3 times (OR 5.80, 95% CI 1.82, 18.44; OR 3.91, 95% CI 2.65, 5.77, respectively) more likely to experience discrimination in a rape crisis or domestic violence center, multiracial/mixed race transgender was almost 3 times (OR 2.98, 95% CI 2.02, 4.40) more likely, Hispanic Latino and American Indian/Alaska Native transgender were about 2 times (OR 2.01, 95% CI 1.18, 3.42; OR 2.09, 95% CI 1.22, 3.55, respectively) more likely to experience discrimination in these centers compared to those who identified as White (Table 4).

Table 5 adjusted simultaneously for all confounding covariates. Always recognized as transgender continued to show significant associations with discrimination in a health-care setting (OR 1.48, 95% CI 1.09, 2.00) and in two individualized health-care settings: social service (rape crisis center, domestic violence center, OR 5.22, 95% CI 2.67, 10.19) and mental health (mental health clinic, drug treatment program, OR 1.87, 95% CI 1.22, 2.88). Those recognized most of the time had 3.09 (95% CI 1.65, 5.67) times the odds of discrimination, sometimes had 2.67 (95% CI 1.50, 4.67) odds and occasionally had 2.18 (95% CI 1.34, 3.56) times the odds of discrimination in the social service settings.

**Table 5:** Multivariable logistic regression analyses for discrimination in health-care settings based on recognizability as transgender among 6106 transgender in the U.S., adjusted for demographic characteristics, experiential and medical factors (Model 4)^a^

	Discrimination in a health-care setting^c^ (*N* = 6106)	Discrimination in rape crisis or domestic violence center (*N* = 3088)	Discrimination in mental health clinic or drug treatment program (*N* = 4249)	Discrimination in hospital/doctor/ambulance or emergency room setting (*N* = 5618)
Characteristic	OR (CI)^b^	OR (CI)^b^	OR (CI)^b^	OR (CI)^b^
People can tell I’m trans/GNC				
Always	1.48* (1.09, 2.00)	5.22* (2.67, 10.19)	1.87* (1.22, 2.88)	1.19 (0.88, 1.62)
Most of the time	1.20 (0.94, 1.53)	3.09* (1.65, 5.67)	1.62* (1.13, 2.32)	1.13 (0.89, 1.43)
Sometimes	0.80 (0.64, 1.00)	2.67* (1.50, 4.76)	1.31 (0.94, 1.83)	0.78* (0.62, 0.97)
Occasionally	0.94 (0.79, 1.13)	2.18* (1.34, 3.56)	1.06 (0.81, 1.39)	0.90 (0.75, 1.07)
Never	1 (ref)	1 (ref)	1 (ref)	1 (ref)
Assigned sex at birth				
Male	1 (ref)	1 (ref)	1 (ref)	1 (ref)
Female	0.88 (0.74, 1.06)	1.10 (0.74, 1.63)	1.01 (0.78, 1.30)	0.89 (0.76, 1.06)
Ethnicity/race				
White	1 (ref)	1 (ref)	1 (ref)	1 (ref)
Black/African American	0.77 (0.55, 1.09)	1.43 (0.80, 2.55)	0.72 (0.45, 1.16)	0.81 (0.57, 1.14)
American Indian/Alaska Native	1.93* (1.44, 2.60)	1.63 (0.90, 2.93)	1.57* (1.08, 2.27)	1.89* (1.43, 2.51)
Hispanic/Latino	1.19 (0.88, 1.61)	1.17 (0.62, 2.20)	1.35 (0.89, 2.05)	1.27 (0.94, 1.72)
Asian Pacific/Islander	0.85 (0.54, 1.32)	0.42 (0.10, 1.80)	1.08 (0.58, 2.03)	0.86 (0.55, 1.33)
Arab/Middle Eastern	1.35 (0.58, 3.16)	3.96* (1.11, 14.05)	1.53 (0.53, 4.46)	1.46 (0.62, 3.40)
Multiracial/mixed race	1.44* (1.15, 1.80)	1.84* (1.19, 2.85)	1.74* (1.30, 2.32)	1.57* (1.26, 1.94)
Education				
Low	0.62* (0.51, 0.76)	1.03 (0.64, 1.64)	0.97 (0.72, 1.29)	0.59* (0.48, 0.71)
Medium	0.75* (0.64, 0.87)	1.08 (0.72, 1.61)	1.07 (0.85, 1.35)	0.72* (0.62, 0.84)
High	1 (ref)	1 (ref)	1 (ref)	1 (ref)
Sex work	2.85* (2.29, 3.55)	5.21* (3.65, 7.43)	2.90* (2.25, 3.73)	2.27* (1.84, 2.81)
Drug sales	1.93* (1.53, 2.44)	1.37 (0.91, 2.08)	1.85* (1.40, 2.45)	1.95* (1.56, 2.44)
Other street economy	2.38* (1.67, 3.40)	2.80* (1.64, 4.78)	2.13* (1.43, 3.16)	2.39* (1.71, 3.34)
Jail/prison	1.27* (1.07, 1.51)	1.31 (0.94, 1.84)	1.51* (1.21, 1.88)	1.20* (1.01, 1.42)
Gender-related mental health diagnosis	2.16* (1.91, 2.44)	1.86* (1.37, 2.51)	2.00* (1.66, 2.41)	2.11* (1.87, 2.38)
Disability	1.16* (1.02, 1.31)	0.86 (0.63, 1.17)	1.11 (0.92, 1.33)	1.17* (1.03, 1.32)
HIV status				
HIV negative	1 (ref)	1 (ref)	1 (ref)	1 (ref)
HIV positive	0.81 (0.52, 1.25)	0.93 (0.49, 1.75)	1.16 (0.70, 1.92)	0.82 (0.53, 1.27)
I don’t know my status	0.89 (0.71, 1.12)	1.04 (0.62, 1.76)	1.17 (0.85, 1.60)	0.94 (0.75, 1.18)

Adjusted for: people can tell I’m trans/GNC, age, sex at birth, ethnicity/race, education, sex work, drug sales, other street economy, jail/prison, gender-related mental health diagnosis, disability, and HIV status

* Significance at .05 level

^a^The total, *N*, varies from variable to variable because of missing data and also exclusion of those who responded as “not applicable” because they have not tried to access the setting

^b^OR odds ratio, CI 95% Confidence Interval

^c^Health-care setting is a combination of all the settings: hospital/doctor, emergency room, ambulance/emergency medical technician, rape crisis center, domestic violence shelter, mental health clinic and drug treatment program

In the fully adjusted model, those who had worked in sex work and other street economy, or received a gender-related mental health diagnosis were significantly more likely to experience discrimination in every individualized health-care setting independent of the other factors (Table 5). In the same model, transgender who had been involved in drug sales, or been to jail/prison, were significantly more likely to experience discrimination in at least two individualized health-care settings irrespective of other exposure factors.

No significant association was found with having a disability and experiencing discrimination in a social service or mental health settings. However, a significant association was found between disability and experiencing discrimination in a hospital/doctor, ambulance, or emergency room setting. Experiencing discrimination in individual health-care settings was also not related to assigned sex at birth or not knowing one’s HIV status.

## Discussion

This study found that being recognized as transgender had a significant effect on perceived discrimination in health-care. Sex work and other street economy, known experiential factors affected by discrimination, were also significantly associated with discrimination in health-care settings. The hypothesis that persons recognizable as transgender face greater discrimination when accessing health-care services was supported.

Although discrimination in the general U.S. population is well documented (Kessler, Mickelson, & Williams, 1999), the findings in this study show transgender who are recognized as transgender, even only occasionally, face discrimination in health-care settings, with more than one-third of transgender participants reporting having experienced discrimination in health-care settings. Moreover, being recognized as transgender was associated with discrimination independently of demographics, experiential and medical factors. For example, crude odds ratios for those identified as Black/African American showed strong significant association with discrimination in a social service setting (rape crisis center and domestic violence center) and a mental health setting (mental health clinic and drug treatment program). However, in the fully adjusted model, no significant association was found, indicating that solely being Black/African American was not associated with discrimination in these social services and mental health settings. The fully adjusted model showed that the level of recognition was a very important determinant of discrimination in a health-care setting, irrespective of other significant predictors such as being involved in sex work, drug sales and other street economy, having been to jail/prison, having had a gender-related mental health diagnosis, and belonging to a certain race/ethnicity. Thus, that others can tell that a person is transgender seems in and of itself to be the apparent basis for discrimination.

Evidence suggests that sex workers, those who have ever been to jail/prison, and HIV positive individuals face greater discrimination than those who have not had these experiences (DFID, 2007; King, Maman, Bowling, & Dudina, 2013). The findings of this study are consistent with this view. Strong associations were found between experiences of discrimination and sex work across all individualized health-care settings, as well as in any health-care setting.

### Strengths and Limitations

Traditionally, cross-sectional studies do not allow for causality to be determined. However, in this study, it is likely to assume being recognized as transgender leads to discrimination and not the other way around. On the other hand, as the measures are self-reported, it is possible that experiencing discrimination in health-care settings could lead to self-awareness with regard to one’s level of recognizability. Moreover, the relationship between discrimination and self-awareness is likely to be complex and bidirectional. Thus, repeated experiences of discrimination might lead to hypervigilance and the expectation of being discriminated again, which in turn might elicit the very rejection that is feared (Hatzenbuehler, 2009).

The composition of the sample who responded to the survey may have contributed to selection bias in that fully 77% (*n* = 4699) were self-identified White participants. This is in keeping with other health surveys, although such a large white population suggests the need to replicate this study with a more racially/ethnically diverse sample if possible (Reisner et al., 2015). In general, higher educated and more affluent people are more likely to participate in surveys (Curtin, Presser, & Singer, 2000; Goyder, Warriner, & Miller, 2002; Singer, van Hoewyk, & Maher, 2000). This study also had a high number of transgender with education greater than a secondary school level (80%). The disproportionately white and well-educated sample was likely due to the use of the primarily internet-based data collection. The very low prevalence rates of HIV (2.4%) compared to recent meta-analyses (e.g., Baral et al., 2013) constitute an additional limitation and may likely be due to sample composition. Given the skewed demographic composition of the sample, it is most probable that there is an underestimation of discrimination, since White people and persons of higher sociodemographic status are generally less likely to be discriminated against compared to their less privileged counterparts (Ren, Amick, & Williams, 1999). Another limitation is the lack of comparative data concerning the percentage of non-transgender persons who would self-report discrimination in these health-care and social service settings.

Bias may also exist due to the use of self-reported survey data. This may be particularly relevant for the primary outcome measure, which was self-reported experience of discrimination. Thus, it is not known whether the discrimination was legally documented. Nevertheless, discrimination is by its nature largely a subjective experience and therefore prone to bias. An unknown factor in the present study is the extent to which persons with multiple non-normative characteristics might have raised expectations concerning discrimination in health-care settings. Despite these limitations, the self-reported nature of the information collected by the survey could be a strength, since sensitive questions were not being asked face-to-face.

Other limitations of this study may be that how long an individual had identified as transgender was not considered. Also, all transgender were included, regardless of whether they considered themselves to be male-to-female (MtF), or female-to-male (FtM), or other types of transgender identities. The distribution of health inequalities differs within different subsets of the transgender community. Distinguishing between various subsets could yield more specific results for the targeted populations. A study in New York City of new diagnoses of HIV infection among transgender people revealed that 95% were transgender women (CDC, 2011). This highlights the importance of looking at specific populations within the transgender community to address their specific risks and health challenges. Also, although the current study acknowledges the notion that transgender persons may have multiple sources of perceived discrimination due to race/ethnicity, medical and disability status, as well as experiential factors, the current analytical approach did not consider ways in which the various categories might have depended upon each other for meaning. Future studies might want to consider a more intersectional approach in recognition of the fact that these categories are experienced simultaneously, rather than as separate entities (Cole, 2009).

All measures in the National Transgender Discrimination Survey were created for the general survey. Lack of validated measures could have resulted in misclassification of information. Yet, despite this possible limitation, the dataset is one of the largest study of discrimination among transgender persons and the comprehensive nature of the data available is unique. Although it is not clear from the data who discriminated against the participants, the above results show a need for all engaged in health-care settings to be aware of discrimination of persons identified as transgender. Such an awareness can encourage health-care providers and others who interact with transgender persons in health-care settings to critically examine attitudes and practices that might create barriers to the delivery of quality health care for transgender persons. The recognition of judgmental attitudes and routines that may be perceived as excluding certain groups is a necessary step toward the creation of a welcoming and gender-affirming environment. Thus, the current results can potentially influence cultural competence trainings and contextualize transgender care. They can also influence policies that create safe and fair opportunities for transgender to access and receive health care.

Moving forward, it is vital that discrimination of transgender in health-care settings is addressed. Mansh, Garcia, and Lunn (2015) suggested that more sexual and gender minority health professionals act as peer-educators and lead the way for equality by fostering diversity and inclusion in medicine. More medical programs and practices could develop recruitment practices that promote competent physicians from among sexual and gender minority health professionals. The need for a modified medical education curricula that raises awareness of transgender discrimination should be addressed (Fallin-Bennett, 2015). Ideally, such awareness should be incorporated early in the medical educational curriculum and could be part of standard clinical training in developing and maintaining a therapeutic patient–physician relationship. In addition, professional development for medical and mental health professionals in health-care settings should also include information on transgender discrimination and ways to combat it and also opportunities for training. A recent study of psychiatrist’s attitudes toward transgender indicated a shift is taking place away from negative attitudes and intolerance of transgender (Ali, Fleisher, & Erickson, 2016). There is hope for eliminating discrimination of transgender in health-care settings.

### Conclusion

Discrimination in health-care settings is pervasive for transgender who are recognized as transgender. Public health efforts to improve access to indiscriminate health care for transgender individuals may benefit from consideration of demographic, experiential, and medical risk factors to more fully understand the source of the seemingly excess risk of discrimination among persons recognized by others as being transgender. Furthermore, to the extent that demographics, experiential factors and medical factors affect discrimination of transgender when accessing health care, interventions to prevent discrimination in health-care settings need to be implemented. This could be through simple awareness campaigns or training to increase gender-affirming care in health-care professionals.

Qualitative research that allows for the experience of transgender in relation to discrimination is valuable. Policy makers need quantitative and qualitative data from multiple sources in order to create public policies that would best meet the needs of transgender individuals.

The effect of discrimination in health-care settings for transgender has not been fully explored in current research. In spite of these knowledge gaps, there is value in sensitizing and training health professionals about discrimination and how to address this in health-care settings.
